# ROS Homeostasis in Abiotic Stress Tolerance in Plants

**DOI:** 10.3390/ijms21155208

**Published:** 2020-07-23

**Authors:** Kalaivani K. Nadarajah

**Affiliations:** Department of Biological Sciences and Biotechnology, Faculty of Science and Technology, Universiti Kebangsaan Malaysia, 43600 UKM BANGI, Malaysia; vani@ukm.edu.my

**Keywords:** antioxidative, enzymatic and non-enzymatic enzymes, ROS reactive genes, hormones, signaling, environmental stresses

## Abstract

Climate change-induced abiotic stress results in crop yield and production losses. These stresses result in changes at the physiological and molecular level that affect the development and growth of the plant. Reactive oxygen species (ROS) is formed at high levels due to abiotic stress within different organelles, leading to cellular damage. Plants have evolved mechanisms to control the production and scavenging of ROS through enzymatic and non-enzymatic antioxidative processes. However, ROS has a dual function in abiotic stresses where, at high levels, they are toxic to cells while the same molecule can function as a signal transducer that activates a local and systemic plant defense response against stress. The effects, perception, signaling, and activation of ROS and their antioxidative responses are elaborated in this review. This review aims to provide a purview of processes involved in ROS homeostasis in plants and to identify genes that are triggered in response to abiotic-induced oxidative stress. This review articulates the importance of these genes and pathways in understanding the mechanism of resistance in plants and the importance of this information in breeding and genetically developing crops for resistance against abiotic stress in plants.

## 1. Introduction 

Abiotic stresses affect plant morphology, biochemistry, physiology, and anatomy through processes such as photosynthesis, respiration, growth, and development, where prolonged stress induces death [1,2,3]. Plants have evolved physiological and metabolic mechanisms that may be instrumental in alleviating environmental stresses such as drought, cold, salinity, metal toxicity, and submergence. These processes are regulated through the activation of gene networks or pathways that result in either enhanced tolerance or resistance [3,4,5]. During stress, harmful by-products that are detrimental to plants are produced. Superoxide radicals, hydrogen peroxide (H_2_O_2_), hydroxyl radicals (OH^•^), and singlet oxygen (^1^O_2_) are reactive oxygen species (ROS) formed in response to the reduction of oxygen molecules *in planta* [5]. These oxygen radicals affect proteins and lipids, resulting in cellular damage and death [6]. When provided with optimal growth conditions, the ROS levels within organelles are low. However, in periods of stress, these levels are elevated due to disturbances in cellular water potential, affecting cellular homeostasis [7,8,9]. Homeostasis of ROS in the cell is achieved through a balance between its production and scavenging [9], where growth conditions, severity, and duration of stress affects cellular equilibrium [10,11]. ROS production and scavenging are somewhat opposed, where an overproduction of this molecule is toxic to the cell, while, as a signal transducer, it triggers the plant’s defense. The initial burst of ROS production activates downstream processes post-stress, which leads to defense mechanism mobilization and the management of stress [12,13,14]. The ROS-scavenging mechanism plays a crucial role in protecting against stress damage in plants [7,14,15]. Understanding the mechanism of ROS production, signaling, and scavenging allows for a powerful strategy to enhance crop tolerance toward environmental conditions [16]. 

ROS is generated in the cell as a consequence of electron leakage during photosynthesis and respiration [17]. To moderate the overproduction of ROS and oxidative stress, plants have a well-regulated antioxidative mechanism that consists of enzymatic and non-enzymatic components that can balance ROS synthesis and scavenging and prevent cellular damage [18,19,20]. Superoxide dismutase (SOD), catalase (CAT), and peroxides (POX) are among the enzymatic antioxidant systems that regulate the homeostasis of ROS within organisms [21]. These enzymes are involved in the reduction process of O^2−^ to H_2_O_2_ [22]. The non-enzymatic components, which are generally made up of players such as ascorbic acids, α-tocopherol, flavanoid, glutathione, carotenoids, lipids, and phenolic compounds, efficiently mitigate oxidative damage by reducing ROS activity or by working together with the enzymatic players to achieve efficient antioxidant activity via the utilization of H_2_O_2_ [19,20]. The mode of action and regulation of both these antioxidant systems and the members will be discussed later.

In the past years, the importance of oxidative stress management and the role of local and systemic ROS signaling in addressing abiotic stress have been extensively studied [15,23]. Despite these studies, there remains a wide variation in the reports on antioxidant activities in various abiotic-stressed plants. Here, we provide an overview of the antioxidant stress management mechanisms in plants and the role they play in abiotic stress response. The regulation and key components of abiotic stress management are yet to be completely understood. This review provides an overview of the status of ROS production in plants and how the plant system achieves ROS homeostasis. Further, the signaling involved in achieving homeostasis between ROS production and scavenging within plant organelles is discussed in brief. Most importantly, this review provides a list of genes involved in ROS regulation in abiotic stress. This information is important for us to identify pathways and genes that regulate oxidative stress in plants and to determine key targets for use in the breeding and genetic engineering of crops.

## 2. Antioxidants and Abiotic Stress Modulation

The whole plant experiences abiotic stress. The stress experienced is then transmitted to distal tissues in the plant through signaling systems that involve hormones or molecules (abscisic acid (ABA), jasmonate salicylic acid, etc.). This, therefore, indicates the importance of stress management in mitigating the effects of stress on the whole plant [24,25,26]. Reviews have focused on ROS metabolism, ROS sensory response, signaling networks [13,27,28,29], and their ability to cross-talk with other molecules in addressing developmental and environmental stresses [27,30,31]. Most reviews highlight the control over stomatal aperture, reduced CO_2_ levels, and photosynthesis as the contributing factor toward elevated ROS levels in plants [17,32,33,34,35]. Elevated levels of abiotic stress-induced ROS may be produced through a reduction in electron transport in the Calvin cycle and a higher electron leakage during photosynthesis in the Mehler reaction. Together, this results in higher respiration and lower photosynthesis and elevated ROS levels in stressed tissues [36]. Both these reactions occur within the chloroplast and, therefore, require these organelles to be robust against ROS, which is achieved through the function of antioxidant enzymes (enzymatic regulators) that quenches ROS activity. In addition, abiotic stresses (salt, heat, and drought) also influence photo-respiration, resulting in hydrogen peroxide (H_2_O_2_) production [17]. Within plant cells, both H_2_O_2_ and O^2−^ function as secondary messengers that control cell death, cell cycle, growth, development, hormone signaling, and stress responses [37]. However, the scavenging systems are not reactive toward hydroxyl radicals and, at high levels, result in deleterious effects to organelles [32,36,38] through lipid peroxidation, which injures membranes and causes damage to biological molecules such as lipids, nucleic acids, and proteins, ultimately resulting in cellular damage and death [39]. 

Crop production is severely impaired as a consequence of overproduction of ROS in the organs during abiotic stresses [21]. In response to endogenous signals (hormones and signal molecules) and exogenous environmental stimuli (biotic or abiotic stresses), several enzymes such as NADPH oxidases, amine oxidases, polyamine oxidases, oxalate oxidases, and a large family of class III peroxidases that are localized at the cell surface or apoplast are induced and result in elevated ROS production [21,40,41]. Therefore, to reduce loss from ROS, the antioxidant activity in the plant needs to increase [42]. As ROS is continuously produced in plants within the chloroplast, peroxisome, and mitochondria, ROS removal systems have to be well-controlled to ensure the wellbeing of tissues and organs. Resistant lines exhibit better ROS removal systems and membrane stability toward deleterious radicals compared to susceptible ones [28,43]. It is, therefore, important for the homeostasis of ROS scavenging to be maintained in all stress induced (biotic or abiotic) and normal physiological responses *in planta* [18,44]. 

## 3. Multi-facetted Mechanisms in Abiotic Stress-Related ROS Homeostasis

In unstressed conditions, ROS is generated within the cell at low levels. However, when subjected to abiotic stresses, ROS levels are elevated, which activates stress pathways within the plant cells [27,45]. Therefore, it is essential that the various enzymatic and non-enzymatic ROS-scavenging systems present in different organelles work together in ROS detox and achieve ROS homeostasis [46,47]. Ford et al. (2011), in a proteomic study conducted on wheat, showed that several antioxidative enzymes were present under stress [48]. The most dominant enzyme was catalase (CAT), which is required for metabolizing photorespiratory H_2_O_2_ when water is limited like in drought. Similar antioxidative systems were also present in *Arabidopsis thaliana* [49] where core genes for redox homeostasis include antioxidative and reductant-regenerating enzymes, which respond in both a complex and specific manner. These genes that regulate enzymatic and non-enzymatic processes work in tandem within the system. As many genes are induced during ROS response, an equal number are repressed during the same process [50,51,52]. 

Besides the enzymatic and non-enzymatic processes, sugars are the new emerging ROS scavengers. The emerging “sugar as antioxidant” concept is based on redox balance that is achieved through their relationship in photosynthesis, mitochondrial respiration, and fatty acid β-oxidation in various organelles. Excess sugar results in increased cytosolic H_2_O_2_ through metabolic pathways while sugars also result in the reduction in power that contribute toward H_2_O_2_ scavenging via the oxidative pentose phosphate (OPP) pathway, which feeds the NADPH metabolism that activates the antioxidative process [53,54]. This is the dual nature of sugars in ROS homeostasis. Sugars like mannitol can protect from the oxidative damage of chloroplasts. These sugars affect gene expression through sugar-specific signaling cascades, which regulate the expression of abiotic stress-related genes such as superoxide oxidase (SOD), heat shock proteins (HSP) and glutathione-S-transferases (GST). In recent years, hexokinase (HXK), Snf1-related kinase 1, and INV have been identified as sugar signaling regulators. Trehalose is another sugar that has been shown to regulate abscisic acid (ABA) metabolism and protect against oxidative stress of photosystem II (PSII) during stress in plants [55,56]. These sugar-specific pathways together with plant hormone signaling and stress-related pathways, when integrated, are able to participate in plant defense responses. 

Reports have also implicated proline in maintaining redox homeostasis by resulting in NADPH utilization. In a study involving transgenic wheat, higher proline resulted in reduced lipid peroxidation and ROS damage during abiotic stresses [57]. Proline mitigates stress through up-regulation of proline biosynthesis, scavenging of OH^•^ or ^1^O_2_, and an active proline metabolic flux linked to other metabolic pathways. Through this, proline maintains cellular energy and NADP^+^/NADPH balance. This protects cells and contributes toward other pathways such as the tricarboxylic acid cycle (TCA) and GSH. Proline feeds into the electron transport chain (ETC) via proline dehydrogenase (PRODH) that results in superoxide and H_2_O_2_ formation. When PRODH expression is increased due to high ROS, apoptosis and cell death results in the activation of the hypersensitive response (HR). PRODH-dependent ROS production in the mitochondria is linked to proline, pyrroline-5-carboxylate reductase (P5CR), and delta-1-pyrroline-5-carboxylate dehydrogenase (P5CDH). Therefore, an increase in PRODH/P5CDH results in an increase in proline metabolic cycling where P5C is converted to proline via P5CR and NADPH [58].

Sharma et al. (2011) stated that proline accumulates during drought as a solute where mutants defective in proline production were sensitive to drought. Pro-Glu is also implicated in NADP[H] homeostasis [59]. Proline and glycine betaine aids in water uptake [60,61] and ROS quenching, which protects against tissue damage [62,63]. Besides scavenging hydroxyl ions, prolines also bind redox active ions and protect against hydroxyl ion damage [64]. Together with sugars, prolines protect the photosystems against peroxidation in drought [65]. Recently, ABA-insensitive mutants revealed that *abi4* increased proline levels in stress and could not be rescued through exogenous application of ABA. However, when sucrose was supplied, the ABA response was restored, indicating that ABI4 has a role in ABA-sugar regulation of proline [53]. Excess ROS generation may also be circumvented through alternative oxidases that divert the electron flow and reduce electron leaks that generate O_2_^•−^ [66]. There are possibly other mechanisms utilized by plants to assist in the balancing of ROS levels and the energy spent in plants. These processes may include events such as leaf curling, leaf movement, and photosynthesis apparatus reassembly [67]. Through the change in the ROS levels, plants can perceive stress and respond accordingly through complex pathways and processes [68]. The easiest way of keeping ROS homeostasis within the plant is to remove the stress on the plant system, thus resulting in a reduction in ROS to levels non-toxic to the plant [12]. 

## 4. Signaling and Control in Abiotic Stress-Associated ROS Homeostasis 

Plant systems activate signaling cascades that trigger downstream components to manage both biotic and abiotic stress. H_2_O_2_ signaling pathways result in the accumulation of protectants that guard against the effects of the cellular redox state and the effect therein. ROS participates in stress signaling through the transduction of signals from mitogen-activated protein kinases (MAPKs) [34,69], which leads to the induction of several pathways, and activation of gene expression downstream. The activated MAPKs signaling cascade adjusts the levels of H_2_O_2_ through the detox antioxidant systems [70,71]. The H_2_O_2_ is the most stable and easily disseminated form of oxidative stress. These molecules act as a switch that enables the messenger to be effective. In addition, the H_2_O_2_ molecules have affinity to thiol groups, which implies a role for this molecule in stress modulation [40,47]. Compared to the animal system, plants have a more modulatory effect on the levels of H_2_O_2_ compared to completely destroying the molecule within the cell [32,47]. This is achieved through the role of antioxidants within the cell that regulates ROS signaling and levels in the host [40,72,73]. Primary or secondary messengers trigger plant-signaling cascades. Some plant hormones like auxins (IAA), abscisic acid (ABA), ethylene (ET), cytokinins (CK), brassinosteroids (BR), gibberellins (GA), jasmonates (JA), and salicylic acid (SA) regulate plant defenses and other biological processes in response to stress [73,74,75]. When under stress, the signal is amplified and stress-related genes are induced in response through signal molecules (ABA, SA, JA, and ET) [73,76,77,78]. 

There is an intricate connection between ROS levels and ABA produced in plants. ABA is probably the most important signal molecule that controls a lot of physiological processes including stress response to abiotic stress [79,80], where it regulates osmotic balance and induces resistance to stresses [79,80,81]. This is achieved through the activation of antioxidant genes (CAT, SOD, peroxidase (POX)) by ABA [82] through ROS-induction and increased levels of NADPH oxidase [83]. In rice, drought-hypersensitive mutants DSM1 and DSM2 have shown the ability to regulate POX expression and control ABA levels that lead to ROS quenching [84,85]. Further, over-expressed *OsCPK4* and *OsSIK1* genes regulate avoidance of lipid peroxidation, and results in the accumulation of SOD, CAT, and POX that acts to lower the H_2_O_2_ levels in the cell [86,87,88]. Up-regulated levels of antioxidant genes (CAT, SOD, POX, GST) result in reduced ROS levels. Research has also shown that the induced expression of GST genes by cold, salt, drought, and heavy metals is a common mechanism for increased tolerance to oxidative damage. In alfalfa, the over-expression of the *MsGSTU8* gene with higher GST activity reduces ROS accumulation by increasing other antioxidant enzyme activities to improve osmotic regulation and reduce ROS damage [89]. Through mutant studies in Arabidopsis, two PP2C phosphatases ABI1 and ABI2 were identified as negative regulators of ABA signaling, and down-regulators of H_2_O_2_ in vitro. ABI1 and ABI2 are involved in Ca^2+^ ion channeling through ABA signaling that controls stomatal closure. ABA-induced Ca^2+^ ion channels were disrupted in *abi1-1* and *abi2-1* mutants, where stomatal closure was affected in the *abi2-1* mutant, while *abi1-1* remained functional. However, in vivo, both genes interacted with GPX3 (glutathione peroxidase) and regulated ABA- and H_2_O_2_-induced stomatal closure. Oxidized GPX3 reduced the phosphatase activity of ABI2 and converted ABI2 to an oxidized form in vitro. ABI1 acts upstream of ROS production and ABI2 works downstream of ROS production in the cell [90,91,92]. In another set of *Arabidopsis* genes, *AtGPX3* and *AtGPX6*, H_2_O_2_ levels affect ABA signaling, ABA-responsive gene expression, CA^2+^ channel activation, and stomatal closure, indicating that the redox status regulates all these processes [93,94,95].

## 5. Organelles Regulation of ROS Homeostasis during Abiotic Stress

**Figure 1:**
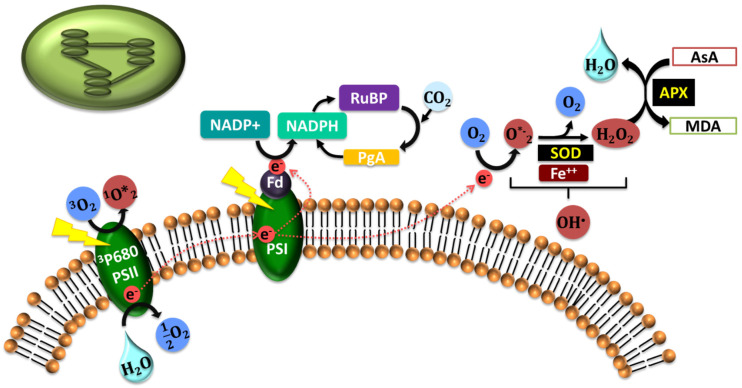
A diagrammatic representation of the processes in the chloroplast during stress where reactive oxygen species (ROS) is produced through inhibition of CO_2_, and low water levels are due to stress. The electron transport chain (ETC) in the photosystem (PS) is the main source of ROS in chloroplast.

**Figure 2:**
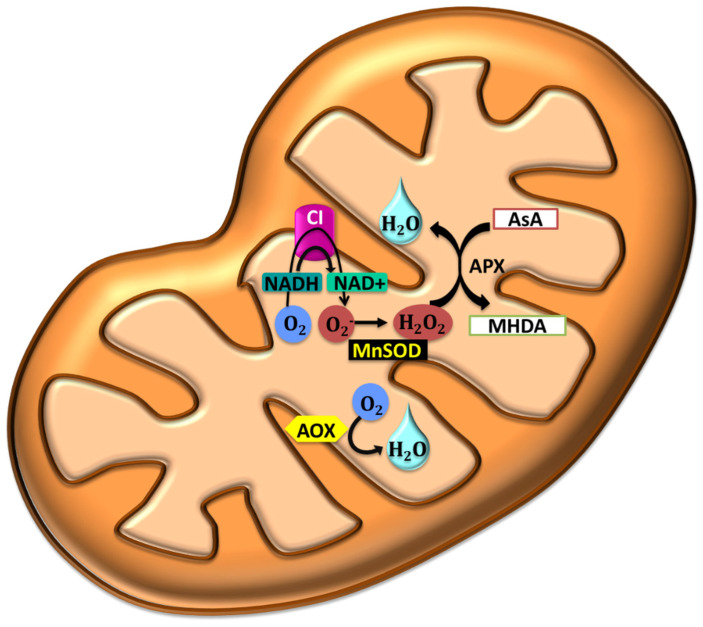
The process of redox in the mitochondria. Any form of stress causes alleviation in ROS due to ATP synthesis, leading to a reduction in the ubiquinone pool (UQ) pool. Several enzymes work together to manage ROS levels in mitochondria.

**Figure 3:**
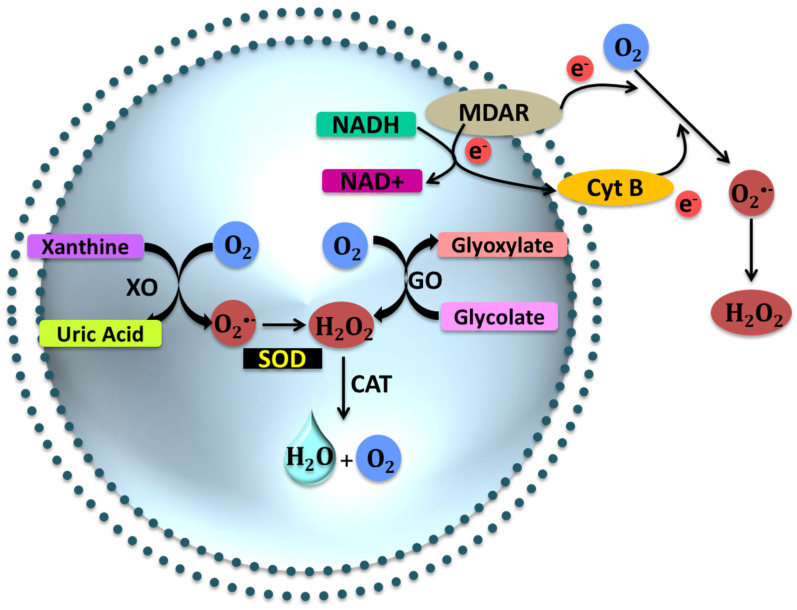
The process of redox in peroxisome during stress. Xanthine oxidases convert xanthine and hypoxanthine to uric acid and O_2_^−^, while the proximal membrane produces O_2_^−^ via NADH and Cytb.

**Figure 4:**
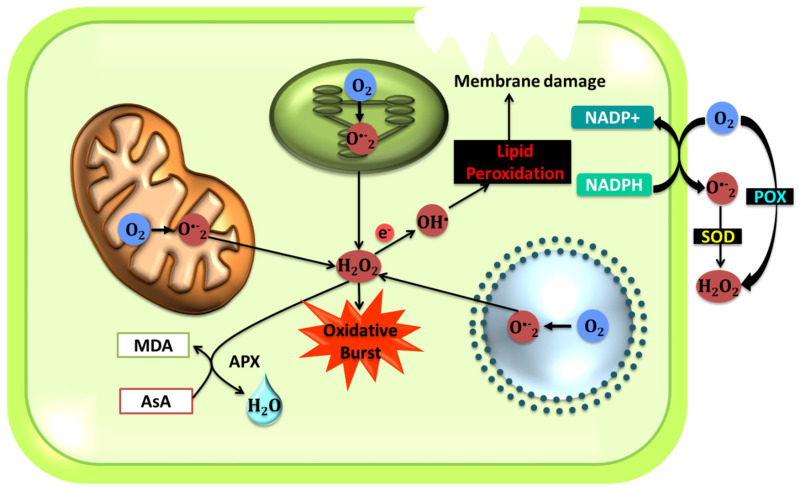
The processes involved in the production and control of ROS in different organelles within a plant system. The above is observed during stress response in plants. All these organelles are collectively responsible at maintaining ROS homeostasis in the cell.

In the following sections, we will review the effect of abiotic stresses on plant cells and their effects on different organelles. Figure 1, Figure 2, Figure 3 and Figure 4 describe the processes ongoing in each organelle under abiotic stress.

### 5.1. ROS Regulation in the Chloroplast 

Photosystems (PS) I and II are the reaction center in the chloroplast where ROS is produced through inhibition of CO_2_, and low water levels are due to stress. Control of ROS levels in the chloroplast is vital for plant survival under stress [96]. The ETC in the PS is the chief source of ROS in chloroplasts. The ROS status in plants changes with the physiological and environmental status of the plant. When not stressed, the electrons flow from the excited PS to reduce NADP to NADPH, which then enters the Calvin cycle where CO_2_ is reduced as the electron acceptor. Conversely, there is an overload on ETC, which results in electron leakage from ferredoxin to O_2_, producing O_2_^•−^ [97]. When exposed to high light intensity with low CO_2_ intake due to stomatal closure, there is a direct transfer of electron to molecular oxygen through the Mehler reaction in PSI [38]. These superoxide radicals are converted by a membrane-bound Cu/ZnSOD to H_2_O_2_, which is further converted by a thylakoid-bound POX to water [98]. The thylakoid-based POX then reacts with thioredoxin to protect against oxidative stress, especially in drought, and provides an alternate water–water cycle to detox the radicals in the chloroplast [18,99,100]. Meanwhile, in PSII, ^1^O_2_ are generated through electron transfer. H_2_O_2_ produced in PSI plays a role in inhibiting ^1^O_2_ through the water–water cycle [101,102,103]. Levels of singlet oxygen when kept unchecked trigger genetic programming such as growth retardation and death through the EXECUTER pathways [104] (Figure 1).

### 5.2. ROS Regulation in the Mitochondria 

The production of ROS is lower in the mitochondria compared to the chloroplast. There is a variation in plant and animal ROS production in mitochondria, wherein in plants, the mitochondria are also the site of photorespiration that is surrounded by carbohydrate solutes [88]. In drought, when respiration rates are high, and transpiration and photosynthesis are low, the demand on mitochondrial ATP is high to compensate for chloroplast ATP production, which causes an increase in ROS levels in the mitochondria [105]. ROS is produced within several sites in mitochondria. In this organelle, the O_2_ is reduced to O_2_^•−^ via the NADPH dehydrogenase [complex I] [106]. Within the mitochondria complex I and III, the electron transfer complex has sufficient energy to reduce O_2_ to ROS from overreduction of the ubiquinone pool (UQ) [107,108,109,110]. An electron is donated to cytochrome C_1_ when UQ is in a fully reduced state. This results in a highly unstable radical complex, which brings about electron leakage and O_2_^•−^ formation [111]. When NAD is low within the complex, there is a reverse electron flow from complex II to I [112,113]

There are alternative ROS-producing sources in the mitochondria like aconitase [114]. While the mitochondria are the source of ROS production, this organelle also has its detoxification system made up of alternative oxidase (AOX) and MnSOD. Aconitase causes the production of ROS while 1-galactono-γ lactone dehydrogenase (GAL) directly donates an electron to the ETC [63]. The O_2_^•−^ that is formed in this organelle is quickly converted to a stable membrane-permeable H_2_O_2_ by MnSOD or ascorbate peroxidase (APX). AOX together with MnSOD work at maintaining the reductive state of the UQ pool while reducing O_2_^−^ to O_2_ [115,116]. Giraud et al. (2008) report that mutant AOX *Arabidopsis* plants were sensitive to drought and light stress, indicating a role for this enzyme in ROS detox [117]. The largest amounts of ROS found in the mitochondria are as O_2_^−^ molecules, which are converted in the detox process by MnSOD and APX to H_2_O_2_ and O_2_ eventually [111]. As in chloroplast, mitochondria produce ROS even in the non-stressed state at basal levels, and any form of stress causes alleviation in ROS due to ATP synthesis, leading to a reduction in the UQ pool [18,116,118,119] (Figure 2).

### 5.3. ROS Regulation at the Peroxisomes

In drought due to reduced CO_2_ and O_2_ levels in the cell, there is an increase in the production of glycolates, which are then oxidized by glycolate oxidase in the peroxisome to H_2_O_2_ [88,120,121]. Just as in the mitochondria and chloroplast, the peroxisome produces the O_2_^•−^ even in their normal metabolism. Through a fine balance between scavenging and production, ROS levels in peroxisome are kept in check [122]. There are two sites of O^2−^ production in the peroxisomal matrix where xanthine oxidases convert xanthine and hypoxanthine to uric acid and O_2_^−^, and at the proximal membrane, O_2_ is used as an electron acceptor by NADH and Cytb to produce O_2_^−^ [123]. Metabolic processes that produce H_2_O_2_ in the peroxisome include β-oxidation, disproportionation of radicals, and the flavin oxidase pathway [88,124,125]. CATs detox the system of H_2_O_2_ while APX and ascorbic acid (AsA)-GSH scavenge H_2_O_2_ in the peroxisome [9,126,127]. The reduced AsA-GSH contents result in lipid peroxidation of peroxisomes. Other than these enzymes, POX, a polyamine-catabolizing enzyme, has been shown to regulate stress-responsive genes to facilitate the production and scavenging of ROS in the peroxisome [128,129,130] (Figure 3).

### 5.4. ROS Regulation in the Apoplast 

Stress-induced ROS production is combined with the effect of ABA in the apoplast [27,131,132]. NADPH oxidases generated in the stomatal guard cells in *Arabidopsis* produces ROS in the apoplast as a consequence of ABA-induced stomatal closure [92,133]. *AtRbohD* and *AtRbohF* are two genes that regulate NADPH oxidases in *Arabidopsis* [134,135]. Other than the NADPH oxidases, peroxidases, cell wall-linked oxidases, polyamine oxidases, and oxalate oxidases play a role in generating H_2_O_2_ in the apoplast [136]. One important source of apoplastic ROS production is the cell wall-linked oxidases [63]. The oxalate oxidase, a cell wall germin-like protein, is known to release H_2_O_2_ and CO_2_ from oxalic acid [137]. This enzyme is mostly involved in plant defense against biotic and abiotic stress. Amine oxidases are found in the apoplast and are contributors to plant defense through the production of H_2_O_2_. These oxidases cause the oxidative deamination of polyamines via cofactors. As observed by Heyno et al. (2011), the hydroxyl ion generation in the apoplastic region of the cell in full or in part contributes to cell wall-bound peroxidases [138]. The increased production of H_2_O_2_ results in higher levels of polyamines and Ca^2+^. This results in more H_2_O_2_ being produced, which, in turn, activates the antioxidative machinery along with increased synthesis of higher polyamines and secondary messengers like Ca^2+^. ABA levels are also elevated, which leads to the activation of polyamine-activated signaling pathways in response to abiotic stresses [128] (Figure 4). 

### 5.5. ROS Regulation at Cell Walls and Plasma Membranes

The cell walls of plants when stressed accumulates oxidative radicals OH^•^, O_2_^•−^, H_2_O_2_, and ^1^O_2_. The cell walls-localized peroxidases, lipoxygenases, oxidases, and polyamines are responsible for the generation of ROS. These oxygen radicals are also responsible for the lipid peroxidation of polyunsaturated fatty acids (PUFA) found in the cell wall of plants that produce ROS [13]. The peroxidases found in the cell walls catalyze the formation of H_2_O_2_ through NADH, through malate dehydrogenase. ROS generation by cell wall-associated peroxidases trigger biotic responses and the alteration of potassium [K] levels in drought-affected plants [88,111]. Liu et al. (2015), in his review, described polyamine’s role in plant stress response as one that is mediated through antioxidant systems or suppression of ROS [12]. Therefore, the exogenous application of polyamines or the activation of polyamine-related genes would lead to the activation of antioxidant processes in situ in plants exposed to drought, salinity, nutrient deficiency, temperatures, and others [18,129,139].

As for the plasma membrane, NADPH oxidases localized in the membrane are responsible for generating O_2_^•−^ through the transfer of an electron from NADPH to O_2_, which is then converted by SOD to H_2_O_2_. The NADPH oxidases in the plasma membranes are crucial players in the stress response of plant cells to environmental factors such as abiotic stresses [18,63]. In certain cases, multiple enzymes were found to catalyze the conversion of O_2_ to O_2_^•−^. For instance, in soybean, other than NADPH oxidase, a quinone reductase also functions in the presence of menadione to facilitate the conversion of O_2_ to O_2_^•−^ [18,63,140] (Figure 4).

## 6. Genes Regulating ROS Homeostasis in Abiotic Stress

**Figure 5:**
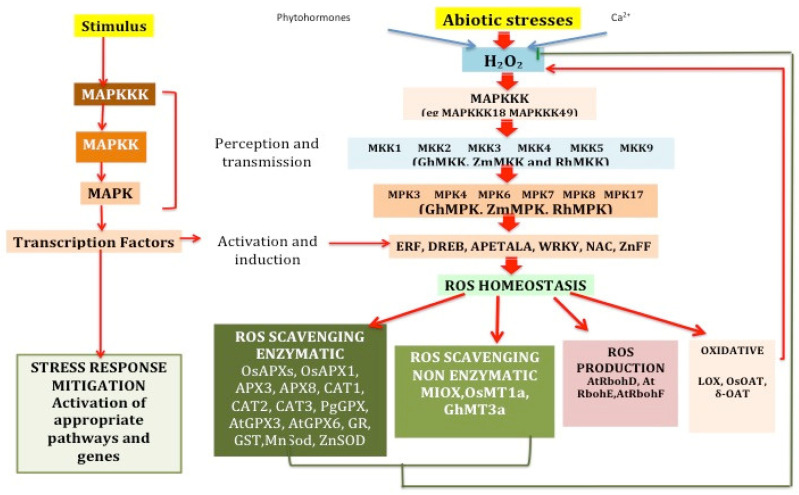
The pathway taken from the time of stimulus to the activation of genes downstream and the genes involved in the regulation of this pathway.

There are an array of genes identified as key players of ROS regulation in plants [79]. Large numbers of genes are activated in response to abiotic stress in plants. These genes are highly regulated in their expression [141]. In the following section, we will briefly expound on the roles played by some of these genes, specifically genes involved in the activation and regulation of ROS in abiotic stress response [142]. These genes have been divided into various classes to enable a systematic illustration [143]. Table 1 and Figure 5 provide details of some of these genes and how these genes are interconnected in the regulation of abiotic stress.

### 6.1. Protein Kinases and Phosphatases 

One of the important gene groups involved in ROS signaling is the mitogen-activated protein kinases (MAPK). There have been many MAPK cascades studied in plants [144]. This gene can be activated by different external stimuli. For instance, MPK6 in *Arabidopsis* is activated by abiotic stress (drought) and repressed under rehydration [145,146]. In roses, RhMPK6 is produced at high levels during hydration, which phosphorylates and stabilizes RhACS1, resulting in ethylene production [147]. Through signal transduction that follows, flower opening and senescence are controlled. Chen et al. (2017) also notes that RhMKK9 is involved in dehydration-dependent ethylene biosynthesis, where it works on RhMPK6-RhACS1 [148]. In another study by Mitula et al. (2015), MAPKKK18 was reported to control stomatal aperture and development [149]. Through mutant studies of this gene, it was concluded that MAPKKK18 controlled ABA-dependent stomatal closure under stress [145,150]. 

GhMKK1 from cotton was able to increase stress resistance and result in ROS homeostasis. Likewise, *BnMKK1* (from *Brassica napus*), when introduced and overexpressed in transgenic tobacco, triggered ABA signaling, causing rapid water loss and drought sensitivity. This, therefore, indicates that this gene generates drought-susceptible plants [151]. GhMKK5, however, reduced tolerance to salt and other stresses [152]. In *Arabidopsis*, overexpression of GhMPK17 resulted in increased H_2_O_2_ levels and osmotic stress [145,153]. In cotton, a novel *GhMAPKKK49* was induced in response to ABA or H_2_O_2_ [142]. Further, GhMAPKKK49 was also hypothesized to interact with GhMKK4 and GhMKK9 in mediating ABA- and H_2_O_2_-mediated abiotic stress responses. Wang et al. (2016) reported that GhMKK3 regulates drought tolerance through control of the water deficit. *GhMKK3* overexpression in tobacco effectively induced ABA-responsive stomatal closure and reduction in stomatal numbers [154,155]. Danquah et al. (2015) noted that GhMKK3 and GhPIP1 work in concert with GhMPK7 to generate drought and ABA-activated MAPK modules [156]. Transgenic tobacco overexpressing *ZmMKK3-1 (ZmMKK3)* from maize exhibited enhanced tolerance toward stress-induced oxidative stress [152]. *ZmMKK1* is induced by ABA in maize roots and results in drought and salinity tolerance in *Arabidopsis* [157]. *ZmMPK4-1*, *ZmMPK7*, and *ZmMPK17* were also involved in the regulation of oxidative stress [158] where *ZmMKK10* is jointly activated by *ZmMPK7* and *ZmMPK3* during drought in maize [159]. Similarly, *AtMPK6, AtMPK7, OsMPK5*, and *OsMPK* show ABA-induced defense as in *ZmMPK3* and *ZmMPK6-2* [152,155]. Salt Intolerance 1 (*SIT1*), a receptor-like kinase (RLK), is expressed at high levels in rice root cells during stress and is induced when exposed to salt stress. SIT1 activation of MPK3 and MPK6 in drought and salinity was determined via immunoprecipitation assays where both kinases were reported to form a complex with SIT1 [160], leading to the phosphorylation of MPK3 and MPK6. From the reports on various plant systems, MAPK pathways are involved in both biotic and abiotic stress modulation and act in concert with phytohormone and calcium ion signaling to activate antioxidant in plants.

Protein phosphatases are another large gene family found in plants. The sequencing project of *Medicago truncatula* identified and characterized PPC2 genes in the genome [161]. Further genome analysis in other plants systems such as rice, tomato, *Arabidopsis*, peppers, and maize indicated the presence of *PP2C* gene families [162,163,164]. These genes are responsible for the plants adaptation to environmental conditions and stresses [165]. There are two main subfamilies of PP2C that are present in plants. The subfamily A of PP2Cs are involved in stress responses that are ABA-dependent, while subfamily B phosphatases are MAPK regulators [165]. Further studies of the PP2C genes in *Arabidopsis* and rice indicate an active role for these genes in abiotic stresses that are ABA-dependent [163,166]. In *M*. *truncatula*, subfamily A genes were either up- or down-regulated during drought in an ABA-contingent manner. Through an in-depth study of the *PP2C* gene in *M. truncatula*, *MtPP2C8*, *MtPP2C37*, *MtPP2C46*, *MtPP2C47*, *MtPP2C67, MtPP2C72*, and *MtPP2C73* were reported to be homologous to the *HAI* PP2Cs in *Arabidopsis* and are induced by stress. In addition, *MtPP2C92* and *MtPP2C65* expression were induced under stress. In *Arabidopsis*, the *HAI* PP2Cs provide drought resistance and have the greatest influence on the ABA-independent low-water-potential phenotypes compared to the classical phenotypes [166]. Further, the homologs of *MtPP2C92* and *MtPP2C65* in *Arabidopsis* (*ABI1* and *ABI2*, respectively) are the most well-documented PP2C genes that are ABA-dependent and are active under abiotic stresses [163,165]. The *MP2C* (homolog with *AP2C1*) in alfalfa acts as a negative regulator of the MAPK pathway in cold and drought [167,168]. The *AP2C2* (homolog *MtPP2C72*) regulates ROS levels in response to biotic and abiotic stresses [168]. *AP2C1* is strongly induced in response to drought and cold, while *AP2C2* is less responsive [164].

### 6.2. Transcriptional Factors

Transcriptional factors (TFs) play an important role in stress-responsive gene regulation and expression. ERF, DREB, APETALA, WRKY, NAC, and Zn finger families play an important role in stress regulation in plants [169,170,171,172]. Zn finger family proteins (ZFP) are important in cellular function in eukaryotes and have been divided into several classes such as C2H2, CCCH, C2HC, and C2C2 based on the location of their Zn residues [173,174]. ZFP have been implicated as regulators of defense, development, growth, and stress in plant systems [175,176]. ZFPs play a key role in oxidative stress response in various plant systems [177]. ZFPs are integral in ROS defense where genes such as *ZAT7*, *ZAT10*, and *ZAT12* are up-regulated in *Arabidopsis* knockout plants during oxidative stress [178,179,180]. A drought- and salt-tolerant *Arabidopsis* mutant, *dst*, encodes a C2H2-type ZFP with the ability to negatively regulate stomatal closure. This is achieved through a DST-mediated H_2_O_2_ regulation of increased stomatal closure and enhanced drought resistance [181]. In rice, the DST complex was reported to work in association with DST Co-activator 1 [DCA1] to regulate stomata, and aperture and downstream regulation of drought-responsive genes. This complex induces genes involved in POX production and H_2_O_2_ detoxification. However, overexpression of DCA1 increased sensitivity to stress [182]. In rice, the *OsAHL1* gene expression improved stress tolerance through alleviation of stress at the plasma membrane [183].

*ZFP36*, another C2H2-type ZFP gene, is involved in antioxidant defense and enhances resistance to oxidative stress tolerance in rice [184,185]. This gene is a major player in the regulation of cross-talk between key players of oxidative stress such as H_2_O_2_, NADPH oxidase, MAPK, and ABA signaling [184]. The incorporation of the *ZFP245* (C2H2) gene in rice increased abiotic stress tolerance through the activation of ROS-scavenging enzymes such as SOD and POX. ZFP179, ZFP182, and ZFP252 were also linked to the oxidative stress response via ROS signaling in rice [27,153,186]. A tandem ZFP, OsTZF1, negatively regulates leaf senescence under drought and oxidative stress in rice through the expression of ROS homeostasis genes and scavenging enzymes [153,187]. Similarly, *GhTZF1*, a TZF gene, also modulates oxidative and senescence stress in cotton through the mediation of ROS equilibrium [188]. 

Another TF family that has a wide involvement in rice and *Arabidopsis* is the WRKY family, which has over 100 WRKY genes in both plants collectively. This TF regulates both biotic and abiotic stresses [189]. The WRKY TF family is recognized by the presence of WRKYGQK heptapeptide and a zinc-finger-like motif at the N- and C-terminus, respectively [190,191]. These conserved domains play an important role in regulating important physiological processes by binding to promoter regions of target genes [161,192,193]. WRKY, in association with ROS and ABA, functions in mitigating the effects of oxidative stress in rice. OsWRKY30 and OsWRKY45 are two WRKY genes in rice that, when overexpressed, are effective at increasing drought tolerance in rice under the regulation of OsNAC6 and SNAC1 as promoters. The OsWRKY45 also plays an important role in abiotic stress in *Arabidopsis* [194,195]. Further, when the WRKY57 gene from *Arabidopsis* was transferred to rice, the transgenic rice showed reduction in water loss, electrolyte leakage, and cell death. In these interactions, OsNAC1 and SNAC1 have been implicated as the promoters controlling WRKY57 function. These plants exhibit up-regulation of stress-responsive genes with higher antioxidant and proline content [196]. In transgenic soybean, the GmWRKY27 interacts with GmMYB17 to repress promoter activity and gene expression of *GmNAC29*, which results in reduced ROS levels and enhanced stress tolerance [197]. GmNAC29 negatively regulates stress where it enhances ROS production enzymes, leading to elevated stress. In another study, Yan et al. (2014) showed that GhWRKY17 reduced drought and salt tolerance in tobacco plants through mediation of cellular ROS levels and ABA signaling [198]. Further, the *BdWRKY36* gene isolated from *Brachypodium distachyon* positively regulates abiotic stress through moderation of ROS homeostasis and regulation of stress-responsive genes [27,199].

NAC (for NAM, ATAF-1,-2, and CUC2) is one of the largest TF families with close to 300 members in just *Arabidopsis* and rice [200,201]. NACs, like the previously discussed proteins, regulate plant growth, development, oxidative stress, and drought tolerance [169,201,202,203,204]. GmNAC2, a soybean NAC, negatively regulates abiotic stress through the induction of ROS signaling and expression of stress-responsive genes [27,205,206,207]. The *Eleusine coracana*-isolated *EcNAC1* gene, when transformed into tobacco, exhibited enhanced ROS scavenging and expression of abiotic stress-related genes [208,209]. Further, the *SNAC3* gene in rice positively regulates stress through ROS homeostasis and enhanced ROS-associated enzyme activity [27,210]. A SNAC1-regulated downstream gene, *OsPP18*, mediates drought resistance via ROS homeostasis [27,211]. The mutant *ospp18* was susceptible to drought and oxidative stress. As the ABA-induced expression of ABA-responsive genes was not disrupted in the *ospp18* mutant, the *OsPP18* gene expression is predicted to be regulated in an ABA-independent manner [27]. 

Another group of transcription factors that regulate multiple abiotic stress responses are the AP2/ERF (APETALA2/ethylene response factor) families, which include DREB/CBF [212]. SUB1A, an ERF found in certain rice varieties, was able to adapt to stress and economize on energies spent in stress through ethylene and gibberellin responsiveness [213]. Following flooding and anoxic injury, plants go through severe desiccation when the water subsides. This results in ROS accumulation in plant tissues [213,214] where SUB1A enhances oxidative stress tolerance through activation of ROS-scavenging genes. *SUB1A* is able to improve plant tolerance to abiotic stress through the induction of ABA responsiveness and activation of stress genes [214,215]. The *JERF3* gene from tomato has been reported to regulate ROS activity and, therefore, reduces the osmotic and oxidative stress response in any abiotic conditions [216] by binding to *cis* elements in stress-responsive genes. This gene in tobacco increased abiotic stress tolerance [216].

### 6.3. ROS-Scavenging and Detoxification Proteins

Under stress, the ROS-scavenging gene families are activated in plants. Here, we present the enzymatic (processes catalyzed by enzymes) and non-enzymatic (non-enzymatic regulation) genes responsible for ROS-scavenging systems that manage the state of detoxification and homeostasis within plant cells. These genes include ascorbate peroxidase (*APX*), CAT, dehydroascorbate reductase (*DHAR*), glutathione peroxidase (*GPX*), glutathione reductase (*GR*), glutathione *S*-transferase (*GST*), monodehydroascorbate reductase (*MDHAR*), myo-inositol monooxygenease (MIOX), peroxiredoxin (*PRX*), proline synthesis, and *SOD*. In a study conducted on transgenic rice, the *MnSOD* gene expressed in chloroplast exhibited a fold increase in antioxidant levels, leading to enhanced stress tolerance. In plant systems, SODs are the frontline defense against ROS and are classified by the metal ions that are bound to their active sites such as copper and zinc (Cu/ZnSOD), manganese (MnSOD), and iron (FeSOD). Different metal ions-bound SODs are found in different cellular locations. For instance, Cu/ZnSOD are found in the cytosol and chloroplasts, while MnSOD is located in the mitochondria and peroxisomes. FeSOD is generally found in chloroplast but is significant in prokaryotes [217]. SODs protect the photosynthetic machinery against ROS in transgenic plants compared to wild type under drought-stress [18,32,99,218]. Similarly, the Zn and Cu-containing superoxides remove oxygen radicals from plant cells. Rice transformed with Zn/Cu SOD genes had increased abiotic stress tolerance [62,219,220]. Further, in transgenic alfalfa, the overexpression of MnSOD from tobacco increased the survival and yield of alfalfa over several seasons in drought conditions [221].

The APX is involved in the initial step of the AsA-GSH cycle that scavenges ROS and protects the plant from stress [219,222]. This haem enzyme scavenges H_2_O_2_ through the AsA-GSH cycle where H_2_O_2_ is converted to water and dehydroascorbate DHA [63,223,224]. In different abiotic stresses, APX activity is elevated [225,226,227]. In rice, several isoforms of the APX gene have been identified in the genome. Out of these isoforms, the *OsAPX2* gene has been shown to protect against oxidative injury in rice seedlings [228,229]. The overexpression of *OsAPX2* in transgenic rice increases APX activity and results in a reduction in H_2_O_2_ and malondialdehyde (MDA) under abiotic stress [229]. The reduction in H_2_O_2_ and malondialdehyde (MDA) is probably the reason behind the increased tolerance exhibited by transgenic rice compared to wild type in the booting stage where spikelet fertility is enhanced [229]. *OsAPX1* exhibited augmented spikelet fertility under cold stress [229]. Prakash et al. (2016) reported that both the *APX3* and *APX8* genes are responsible for drought tolerance in IR64 and Nagina [230]. Like APX, GPX is another large family of diverse peroxidase isozymes without the haem-thiol group. This enzyme is responsible for the reduction of H_2_O_2_ to water, and lipid hydroperoxides to alcohol, hence repairing lipid peroxidation and membrane damage [120,231]. Both the intracellular and extracellular forms of this enzyme are involved in H_2_O_2_ scavenging. GPX and other peroxidase enzymes bound to plant cell walls can oxidize phenols to result in the lignification of cell walls [46]. GPX also functions as an oxidative signal transducer in plants [232]. In a functional study of the *PgGPX* (*Pennisetum glauccum* GPX) genes introduced into rice, the transgenic lines showed an enhanced antioxidant defense via lower H_2_O_2_ and MDA content during abiotic stresses [233].

As a haem-containing enzyme, catalase scavenges H_2_O_2_ in the peroxisome, glyoxysomes, and other organelles during stress-related processes [18,234]. CATs respond variably to different abiotic stresses [225,227,235,236,237]. It has a higher turnover rate minus the requirement for a reductant like APX, giving it a higher affinity and scavenging ability of H_2_O_2_. The catalase isoforms are divided into classes, which are specific to location. CAT1 and 2 are in the peroxisomes and cytosol while CAT3 is in the mitochondria [68]. GR, another oxidoreductase, regulates the GSH redox state by catalyzing the disulphide bond formation of the GSSG pool via NADPH. This is important in maintaining the GSH at a reduced state. GR is predominantly located in the chloroplast but is also reported in cytosol and mitochondria. Both GR and GSH have been identified as enzymes linked with tolerance against various stresses in plants [238,239,240].

MDHAR accompanies APX and scavenges H_2_O_2_ in the mitochondria and peroxisome [236,237]. MDHAR accepts electrons from NADH and, together with DHAR and GR, regulates abiotic stress in rice. DHAR works on ascorbate and recycles ascorbic acid (AsA) [241,242]. AsA is then oxidized to form MDHA, which is further converted to DHA. DHAR through GSH reduces DHA to AsA and undergoes rapid regeneration. This regeneration of AsA is moderated through the NADPH-MDHAR cycle [243], which is essential in ensuring and maintaining a reduced pool of AsA [244,245]. Maintenance of the cellular redox state of AsA is essential in abiotic stress tolerance. Increased DHAR and MDHAR activity was reported in various plants subjected to abiotic stress [99,238,246,247]. GST together with GSH can reduce POX activity in the cell through scavenging. In addition to their ability to conjugate electrophilic compounds to GSH, GST displays POX-like activities [248]. There are over 100 GST genes that have been reported in soybean, maize, and *Arabidopsis* [233] with diverse function, including cellular metabolism, hormone homeostasis, cellular detoxification, apoptosis, and various other biotic and abiotic stresses [249,250]. This enzyme is induced at high levels when plants are subject to abiotic stresses [99,146,251,252].

MIOX produces AsA, which results in antioxidant defense. Oxidative damage in rice is reduced through the overexpression of *OsMIOX* and increased ROS scavenging [19,253]. Another enzyme that scavenges ROS is ornithine δ–aminotransferase (δ-OAT), which synthesizes proline, a non-enzymatic ROS-scavenging system effective in abiotic conditions [21,254]. You et al. (2012) reported that the overexpression of *OsOAT* induced enzyme activities that resulted in ROS quenching and improved antioxidative activity within the plant cells [255]. Other than proline, metallothioneins (MTs) are another low-molecular-weight protein that has metal-binding capabilities. MTs are involved in ROS detoxification and the maintenance of safe redox levels. *OsMT1a* expression in rice is enhanced in plants that are subjected to Zn^2+^ treatment and drought [256]. These plants exhibit elevated levels of APX, CAT, and POD that result in these transgenic lines exhibiting heightened tolerance to drought stress. Zn^2+^ homeostasis is important in improved resistance in plants. OsMT1a interacts with Zn finger transcription factors that can moderate the levels of Zn^2+^ within the cell. In cotton, *GhMT3a* resulted in an enhanced ability to bind metal ions, resulting in efficient ROS scavenging *in planta*. *GhMT3a*, when introduced into tobacco, resulted in improved resistance against multiple abiotic stresses through lower H_2_O_2_ levels than those exhibited in wild-type plants [27,57,100,257]. 

In addition to the enzymatic and non-enzymatic genes that have been mentioned above, sugars are suggested to have a role in antioxidative responses. Fructans, disaccharide, and sugar alcohols possess antioxidative abilities and can efficiently remove hydroxyl radicals within plant cells and organelles [54,258]. This non-enzymatic removal of the °OH radical is important as there are not any enzymatic systems available for the removal of this toxic compound [259]. Galactinol and raffinose have demonstrated similar potential to GSH in the removal of hydroxyl ions from plant cells [260]. The levels of these sugars in chloroplast are comparable to AsA and GSH, suggesting that these sugars play a vital role in scavenging radical ions in this organelle [111,239,261]. However, the role of sucrose as antioxidants is only relevant in plants with high levels of sucrose such as beet and sugarcane, where they have been reported to remove °OH effectively [262].

### 6.4. Other Proteins

#### 6.4.1. Ca^2+^ Transporters and Binding Proteins 

Most of the Ca^2+^-binding sites contain the EF-hand motifs that are highly conserved amongst eukaryotes [263]. The EF-hand motifs are also found in the proteins family that transport and manage intracellular Ca^2+^ concentrations [264]. The influx and efflux of Ca^2+^ across the membranes is achieved through Ca^2+^ATPases or antiporters. Both the calcium transporters and binding proteins help to regulate processes by the altering of Ca^2+^ levels in cells. The main players of calcium binding in plants are calmodulin [CaM], CaM-like proteins (CML), calcineurin-B-like proteins (CBL), and Ca^2+^ dependent protein kinases (CDPK). CaM, CML, and CBL are the sensors, while CDPK are sensor responders that activate the kinase activity of this protein [265]. As drought affects growth, development, and stress tolerance, Ca^2+^ is implicated in the regulation of signaling involving drought-affected processes. Calcium-binding proteins bind calcium and thence activate the downstream phosphorylation cascade of gene expression. This increases the levels of Ca^2+^ perceived by calmodulin and CDPK. *OsACA6*, a form of Ca^2+^ATPase in rice, when overexpressed, is able to moderate the reduction in ROS levels. The overexpression of this gene results in cellular homeostasis through the modulation of ROS-scavenging systems [73,266]. 

Further, another group of proteins, annexins, are implicated in the response toward environmental stresses on growth and development. One such annexin, OsANN1, functions as an ATPase with the ability to bind Ca^2+^ and regulate the inflow and efflux of Ca^2+^ ions. Through the interaction with *OsCDPK10*, a protein kinase, *OsANN1* confers abiotic stress tolerance via antioxidant accumulation [267]. Further, through yeast two-hybrid system analyses, it was demonstrated that OsANN1 interacts with OsCDPK24 and, therefore, regulates abiotic stress responses [268]. The RNAi knockout mutant of this gene was sensitive to drought, while the overexpressing lines showed improved growth and higher expression of the gene under abiotic stress, leading to SOD and CAT activities that facilitate ROS homeostasis through a OsANN1 and H_2_O_2_ feedback mechanism. 

In another study involving stomatal guard cells, it was reported that cytosolic Ca^2+^ increase and activation of ABA results in activation of the anion channel, which causes the plasma membrane of the guard cells to close. In *Arabidopsis*, an ABC protein, AtMRP5 was bound to the plasma membrane of guard cells, which affects the ABA and cytosolic Ca^2+^ levels in the cell. Mutants of this gene showed loss of ability to keep the stomata closed in drought. These mutants also showed impaired ABA activity, indicating that this gene was responsible for Ca^2+^ control over guard cell aperture [269]. 

#### 6.4.2. SRO Proteins

SRO is a plant-specific protein group that has the PARP, RST and WWE domain [270]. The *rcd1* [*radical-induced cell death1*] in *Arabidopsis* exhibited the ability to respond to various stimuli such as ROS stress, salt stress, and irradiation by interacting with numerous transcription factors that facilitate their involvement in developmental and stress-related responses [27,271,272]. The *RCD1* gene regulates signaling pathways that are responsible for quantitative changes to gene expression in response to ROS [273]. *OsSRO1c* is targeted by *SNAC1* in rice [78], where it is induced in the guard cells during abiotic stress and results in the accumulation of H_2_O_2_ and decreased stomatal aperture and water loss. Due to its involvement in stomatal aperture control and water loss, the overexpression of *OsSRO1c* has also been implicated in abiotic stress tolerance of rice through the regulation of the SNAC1 novel pathway and DST [78]. Just like *OsSRO1c*, *OsNAC5* and *ONAC095* enhance drought and oxidative stress tolerance in rice [274]. Further studies of the SRO protein in wheat showed that this gene was also involved in the regulation of salinity stress in addition to redox homeostasis [27]. The regulation of salinity tolerance in wheat is achieved through the point mutation of the Ta-*sro1* allele. The overexpression of the *Ta-sro1* results in regulation of ROS through ROS-associated enzymes such as AsA-GSH and GPX that result in cellular homeostasis [27].

#### 6.4.3. ABA Metabolism-Related Proteins

ABA is involved in the response to abiotic stresses [275]. In drought, *dsm2* mutants have shown impaired β-carotene hydroxylase synthesis in rice [85]. This particular hydroxylase is a precursor of ABA and is inhibited under drought stress. However, in overexpressing DSM2 lines, this gene is expressed at high levels, leading to enhanced resistance to abiotic and oxidative stress. Other than β-carotene hydroxylase, *OsABA8ox3* is another hydroxylase-encoding gene that is involved in ABA catabolism and regulates oxidative stress under various abiotic stresses [27]. RNAi-generated plants of this gene showed improved drought and oxidative stress tolerance with enhanced superoxide dismutase and catalase activities. In another study, transgenic tobacco carrying the 9-*cis*-epoxy carotenoid dioxygenase gene showed enhanced tolerance to abiotic stresses. This enhanced resistance has been linked to production of H_2_O_2_ that induces the expression of ROS-scavenging enzymes [276]. 

## 7. Conclusions and Future Prospective

Abiotic stresses hamper growth and development, which eventually results in low yields and productivity. Stressed plants exhibiting elevated intracellular and extracellular ROS are different organelles, leading to oxidative stress. Though the compartmentalization of antioxidant activities is well defined, the recognition, response, and balancing of ROS activity in the plant require further exploration. The coordination of the different enzymes in different compartments and the regulation of ROS levels in response to stress are questions that require further attention. The ambiguities and gaps in our knowledge are further compounded by the short half-life and the reactive nature of the molecule. From the various studies that have been conducted over the past two decades, we may conclude, in general, that ROS equilibrium involves cross-talk between ABA, Ca^2+^, and various other hormones and signaling molecules. ROS as a signal transducer also activates a cascade of genes that assist in abiotic stress tolerance in a ROS-dependent manner. Genes such as protein kinases and transcription factors are important upstream components that are responsible for the activation of other downstream genes involved in alleviating ROS toxicity. Genes that are involved in the regulation of ROS have been studied quite well in rice and *Arabidopsis*. 

In this review, we have provided an overview of the oxidative and non-oxidative mechanisms involved in the reduction in ROS damage and the provision of tolerance and adaptation to abiotic stress. We are still not completely clear on the mechanism by which Ca^2+^, hormones, and signal molecules regulate abiotic stresses. Perhaps with the development of better imaging systems, we may be able to utilize ion markers that provide a better understanding of their role in ROS metabolism. Further, genome information has been utilized in functional and metabolome studies that provide a clearer view of the ROS network and its reactions. A combination of transcriptome, proteome, and metabolome approaches may provide a comprehensive understanding of the networks involved in ROS production, signaling, and control. These studies may result in the identification of key pathways, regulators, and genes that are responsible for ROS homeostasis in plants. Some of these genes may be developed into biomarkers to be used in plant stress-response studies. 

By understanding the genes and their expression, we are then able to manipulate the endogenous ROS levels to generate plants with improved defense, growth, development, and survival in adverse abiotic stress conditions. Most of the genes identified in ROS homeostasis have been characterized through the generation of transgenic plants. Some of these transgenic lines with overexpressing genes have shown enhanced tolerance to multiple stresses [277]. However, the networks involved in the function of these genes achieving ROS homeostasis requires further investigation and addition of any new and relevant information into the existing pathways. Some of these genes have already been used in elite cultivars and are candidates for biomarkers in the selection of abiotic-resistant crops. The location of these genes and QTLs associated with these genes is also a suitable candidate for use in the breeding and genetic engineering of resistant cultivars. 

## Figures and Tables

**Table 1:** List of mitogen-activated protein (MAP) kinases and the processes they are implicated to regulate.

MAP Kinase	Phosphorylated Amino Acids	List of MAP Kinases	These MAP Kinases Respond or are Involved in These Processes
MAPKKK	Serine Threonine	MEKK1, MEKK2, MEKK3, MEKK4, *MAPKKK18*, *GhMAPKKK49* *DSM1*, *DSM2*	Influences oxidative, abiotic, and biotic stress. Hormones: Abscisic acid;
MAPKK	Threonine/tyrosine	MKK1, MKK2, MKK6, GhMKK1,	Influences oxidative, abiotic, and biotic stresses and cell division. Hormones: Salicylic acid;
		MKK3, GhMKK3,	Influences oxidative, abiotic, and biotic stresses and cell division. Hormones: Salicylic acid;
		MKK4, MKK5 GhMKK4, GhMKK5,	Influences oxidative, abiotic, and biotic stresses Hormones: Jasmonic acid.
		MKK7, MKK8, MKK9, MKK10, RhMKK9, GhMKK9 ZmMKK10	Influences oxidative and biotic stresses, Hormones: Ethylene
MAPK	Serine/Threonine/ Tyrosine	MPK3, MPK6, MPK10 OsMPK6, ZmMPK3, RhMPK6, ZmMPK6-2, OsMPK3, ZmMPK3	Influences oxidative, abiotic, and biotic stresses. Hormones: Jasmonic acid and ethylene
		MPK4, MPK5, MPK11, MPK12, MPK13 OsMPK4ZmMPK4-1, OsMPK5, OsMPK5, ZmMPK5	Influences oxidative, abiotic, and biotic stresses and cell division. Hormones: Salicylic acid;
		MPK1, MPK2, MPK7, MPK14, ZmMPK7, OsMPK2AtMPK7, OsMPK7, GhMPK7	Influences oxidative, abiotic, and biotic stresses. Circadian-rhythm-regulated. Hormones: Jasmonic acid, abscisic acid.
		MPK8, MPK9, MPK15/16/17/18/19/20 GhMPK17, ZmMPK17	Influences oxidative, abiotic, and biotic stresses. Hormones: Jasmonic acid
